# Does the addition of a supportive chatbot promote user engagement with a smoking cessation app? An experimental study

**DOI:** 10.1177/2055207619880676

**Published:** 2019-09-30

**Authors:** Olga Perski, David Crane, Emma Beard, Jamie Brown

**Affiliations:** Department of Behavioural Science and Health, University College London, UK

**Keywords:** Chatbot, engagement, smoking cessation, smartphone apps, mHealth

## Abstract

**Objective:**

The objective of this study was to assess whether a version of the Smoke Free app with a supportive chatbot powered by artificial intelligence (versus a version without the chatbot) led to increased engagement and short-term quit success.

**Methods:**

Daily or non-daily smokers aged ≥18 years who purchased the ‘pro’ version of the app and set a quit date were randomly assigned (unequal allocation) to receive the app with or without the chatbot. The outcomes were engagement (i.e. total number of logins over the study period) and self-reported abstinence at a one-month follow-up. Unadjusted and adjusted negative binomial and logistic regression models were fitted to estimate incidence rate ratios (IRRs) and odds ratios (ORs) for the associations of interest.

**Results:**

A total of 57,214 smokers were included (intervention: 9.3% (5339); control: 90.7% (51,875). The app with the chatbot compared with the standard version led to a 101% increase in engagement (IRR_adj_ = 2.01, 95% confidence interval **(**CI) = 1.92–2.11, *p* < .001). The one-month follow-up rate was 10.6% (intervention: 19.9% (1,061/5,339); control: 9.7% (5,050/51,875). Smokers allocated to the intervention had greater odds of quit success (missing equals smoking: 844/5,339 *vs*. 3,704/51,875, OR_adj_ = 2.38, 95% CI = 2.19–2.58, *p* < .001; follow-up only: 844/1,061 *vs*. 3,704/5,050, OR_adj_ = 1.36, 95% CI = 1.16–1.61, *p* < .001).

**Conclusion:**

The addition of a supportive chatbot to a popular smoking cessation app more than doubled user engagement. In view of very low follow-up rates, there is low quality evidence that the addition also increased self-reported smoking cessation.

## Introduction

Cigarette smoking is one of the leading causes of premature morbidity and mortality with seven million people globally dying of a smoking-related disease every year.^1^ In England, ∼15% of the population are cigarette smokers,^2^ but there is large variation across countries. Supporting smokers to make a successful quit attempt is a public health priority.^3^ About 40% of smokers make a quit attempt each year,^4^ the majority of which are unaided,^5,6^ with ∼15% of those making a quit attempt stopping successfully.^2^ The use of pharmacological and behavioural support, either alone or in combination, can substantially improve the chances of quitting.^7–9^ Although behavioural support delivered face-to-face by trained healthcare professionals is both effective and cost-effective,^10^ specialist stop smoking services in the United Kingdom (UK) and elsewhere are facing substantial funding cuts^11^ and are relatively rarely used.^12^ Internet access and personal smartphone ownership have grown rapidly in the last decade, with 77% of UK adults using a mobile device to access the internet in 2018.^13^ Alongside this rapid growth, a range of digital interventions for smoking cessation have been developed (e.g. websites, smartphone applications or ‘apps’), which have the potential for wide reach at low cost per user. Although digital smoking cessation interventions can help smokers quit,^14^ user engagement tends to be low on average.^15^ Low engagement might be problematic for digital interventions as rates of engagement are positively associated with quitting success,^16,17^ indicating that engagement may be a key mediator of intervention effectiveness. In light of these observations, identifying intervention content and design features (e.g. interactivity, tailoring) that promote engagement with digital interventions is therefore a research priority.^15^ The evidence-informed Smoke Free app (www.smokefreeapp.com) has a large user base with approximately 3,000 new downloads per day, and therefore acts as a useful test bed. The present study used an experimental design to examine whether the provision of a supportive chatbot within the Smoke Free app, powered by artificial intelligence (AI), leads to increased user engagement and quitting success at a one-month follow-up compared with a version of the Smoke Free app without the chatbot.

Engagement with digital interventions can be defined as: i) the extent of use (e.g. amount, depth, duration and frequency of use) and ii) a subjective experience with cognitive and emotional dimensions (e.g. attention, interest and affect).^18^ The problem of low engagement has been observed in controlled trials of digital interventions developed by both academic and industry professionals.^15,19^ Whether or not users engage with a given digital intervention depends on its content, how that content is delivered (e.g. design features), the context in which the intervention is used, and whether or not the intervention succeeds in changing key ‘mechanisms of action’ that mediate successful behaviour change (e.g. motivation, supportive accountability).^18^ When consulted about what features are judged to be important for engagement with smoking cessation apps, potential users have highlighted a desire for features that foster a sense of personal relevance and enhance motivation not to smoke.^20^

**Figure 1.**
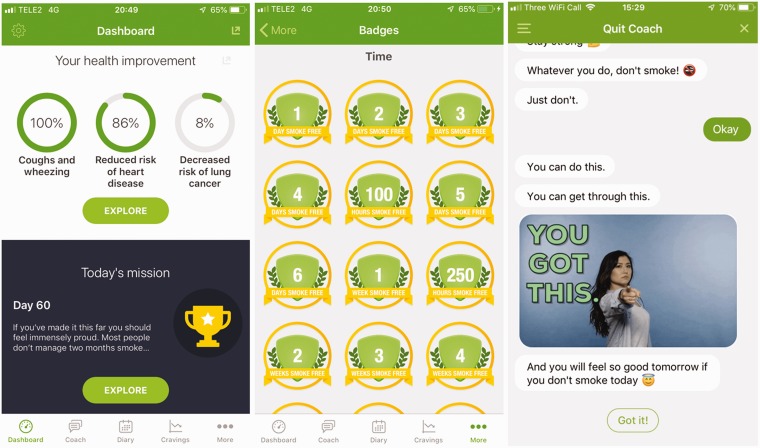
Screenshots of the Smoke Free app.

The Smoke Free app includes behaviour change techniques that research suggests are likely to improve the chances of quitting.^21^ See Figure 1 for screenshots and Supplementary Material File 1 online for a list of behaviour change techniques included in the Smoke Free app. The app guides users through the first month of their quit attempt by helping them maintain their resolve and manage cravings by setting a clear goal, monitoring their progress towards that goal and becoming aware of health and financial benefits achieved to date. It contains several components: 1) a calculator that tracks the total amount of money not spent on buying cigarettes, the number of cigarettes not smoked, the amount of time elapsed since stopping smoking and health improvements expected since the start of the quit attempt; 2) a scoreboard that awards virtual badges (i.e. rewards) for not smoking; 3) a diary which tracks the frequency, strength and location of cravings to smoke; and 4) a graph which displays the frequency, strength and location of cravings to smoke. The paid version of the app (i.e. the ‘pro’ version) also contains daily missions which are assigned from the start of a user’s quit date for two calendar months. In an exploratory randomised controlled trial (RCT) with >28,000 participants, users who were given access to the daily missions for one calendar month were almost twice as likely to remain smoke free at a three-month follow-up compared with users who were allocated to a version of the app without the daily missions.^22^

Chatbots, also known as conversational agents, are computer programs that have conversations with users via auditory or textual media. Recently, a new AI-powered, text-based chatbot was added to the ‘pro’ version of the Smoke Free app (see Figure 1 for screenshots). A key motivation for this was to promote user engagement. According to Mohr’s ‘Model of Supportive Accountability’, the addition of human support promotes engagement with digital interventions through fostering a sense of accountability to a trustworthy, benevolent and competent coach.^23^ As such, AI-driven, automated chatbots intend to mimic the support provided by healthcare professionals. Although the promise of voice- or text-enabled chatbots for promoting engagement with digital tools has been highlighted in the literature,^24,25^ empirical evaluations are scarce at present. A scoping review of conversational agents in mental health interventions concluded that these can help improve engagement and satisfaction, but did not quantify their effects.^26^ In a recent RCT of ‘Woebot’, a conversational agent designed to support young adults with symptoms of depression and anxiety, users allocated to the intervention arm engaged with the chatbot an average of 12 times over the two-week study period.^27^ However, as users in the control group did not have access to an interactive app/website, this study did not provide an opportunity to quantify the added effect of a conversational agent on user engagement. Qualitative studies and single-arm evaluations of stand-alone or embedded conversational chatbots within smoking-related apps indicate that smokers hold positive attitudes towards and engage frequently with these novel features, but are limited by not including a control group.^28,29^ A micro-randomised trial evaluating the effectiveness of a text-based chatbot embedded within a physical activity app is currently underway, which will contribute to the evidence-base.^30^ Hence, due to the lack of studies with a control condition, we currently know little about the added effect of a supportive chatbot on user engagement and quitting success within existing smoking cessation apps.

The present study therefore aimed to answer the following research questions:

1. Do smokers who purchase the ‘pro’ version of the Smoke Free app and are randomly offered the addition of a supportive chatbot [intervention] engage more frequently compared with smokers who are offered the standard version of the app [control]?
2. Do smokers who are randomly offered the supportive chatbot have greater odds of being abstinent at a one-month follow-up compared with smokers who are offered the standard version of the app?

## Methods

### Study design

This was an experimental study with smokers randomised to the intervention and control arms in a planned, unequal ratio of 1:4, using simple randomisation. The app generated a random number between 1 and 100 for each user, with those receiving a number of 20 or below allocated to the intervention arm. The randomisation ratio was selected for pragmatic reasons. The Smoke Free app is currently live on commercial app stores (e.g. Apple App Store). Any novel feature is randomly offered only to a small proportion of users to ensure that it does not have any negative effects prior to roll-out across all users. The analysis plan, but not the experimental design, was pre-registered on the Open Science Framework (https://osf.io/q4kje). Recruitment had finished at the point our analysis plan was registered.

### Eligibility criteria

Smokers were eligible to take part if they: i) owned an iPhone; ii) purchased the ‘pro’ version of the Smoke Free app between 1 September 2018 and 18 December 2018; iii) had their phone set to English language; iv) were aged ≥18 years; v) reported being a daily or non-daily smoker at the time of registration; and vi) set a quit date <2 days before and <14 days after their date of registration. If users registered more than once on the same device (as identified by a unique user ID), data from the first registration were used.

### Measures and procedure

After purchasing the ‘pro’ version of the app and consenting to take part in the study, users were randomised to the study arms. Next, they provided information on time to first cigarette (i.e. <5 min, 5–30 min, 31–60 min, >60 min) and cigarettes per day (CPD). Users were then requested to record their target quit date, which could be any date in the past or future (with those having already quit and those setting a quit date too far in the future being excluded from the present study).

To address the first research question, the outcome variable of interest was the total frequency of engagement, operationalised as the automatically recorded number of logins between the date of registration and the one-month follow-up survey. Although users’ subjective experience (e.g. attention, interest) is also thought to be a key dimension of digital engagement,^18^ the Smoke Free app currently collects data only on the frequency of behavioural engagement. A new login was defined as a new screen record after at least 30 minutes of inactivity.^31^ The predictor variable was group allocation (i.e. intervention *vs*. control). Covariates were time to first cigarette and CPD.

To address the second research question, the outcome variable of interest was self-reported continuous abstinence at the one-month follow-up. The app sends users a push notification one month after their quit date asking them to open the app and respond to a brief survey. No reminders were sent. The survey asks: ‘Have you smoked at all in the last month?’ Response options were: 1) ‘No, not a puff’, 2) ‘1–5 cigarettes’, or 3) ‘More than 5 cigarettes’. Those who respond ‘No, not a puff’ were considered to be abstinent. On the basis of the intention-to-treat principle, those who did not respond to the follow-up survey were retained in the analyses and classified as continuing smokers (i.e. ‘missing equals smoking’ (MES)).^32^ The predictor variable was group allocation (i.e. intervention *vs*. control). Covariates were time to first cigarette and CPD.

### Intervention

#### Control

The ‘pro’ version of the Smoke Free app takes smokers through the first month of their quit attempt and contains: 1) a calculator which tracks the total amount of money not spent on buying cigarettes and the number of cigarettes not smoked; 2) a calendar which tracks the amount of time elapsed since cessation; 3) a scoreboard which awards virtual ‘badges’ to users for not smoking; 4) progress indicators which inform users of the health improvements made since the start of their quit attempt (e.g. pulse rate, oxygen levels, carbon monoxide levels); 5) a diary which tracks the frequency, strength and location of cravings to smoke; 6) a graph which displays the frequency, strength and location of cravings to smoke; and 7) daily missions which are assigned from the start of a user’s quit date for one calendar month.

#### Intervention

In addition to the content provided to users in the control group, users in the intervention group received access to the supportive, AI-driven chatbot. The chatbot was designed to check in with its users twice per day by way of a notification during the first month of a user’s quit attempt and is available for on-demand support as and when needed. Hence, the chatbot was not reliant on the app being opened on users’ phones. The chatbot guides users through the UK Stop Smoking Services’ standard smoking cessation programme (http://www.ncsct.co.uk/) with a friendly, knowledgeable tone of voice. It positively reinforces smoke free days, cravings resisted and quit milestones. Beyond improved engagement, the chatbot was also designed to boost motivation to remain smoke free, reduce cravings and withdrawal symptoms, and improve skills for coping with difficult situations. See Supplementary File 1 for an overview of the behaviour change techniques present in the intervention and control versions of the Smoke Free app, coded against a 44-item taxonomy of behaviour change techniques in individual behavioural support for smoking cessation.^21^

### Ethical approval

The study was approved by UCL’s Research Ethics Committee (Project ID: CEHP/2016/556). Participants were informed that the app was used in an evaluation and asked for permission to use their data for research purposes. Participants in the control group were not made aware of the chatbot at the time of the study.

### Data analysis

All analyses were conducted in R v. 3.5.1.

#### A priori power analysis

In two separate trials of Web- and app-based smoking cessation interventions, users who logged in a median of eight times or more had increased odds of quitting success.^16,33^ Prior to implementing the chatbot (i.e. between 1 January 2018 and 31 May 2018), Smoke Free users logged in a median of seven times. Hence, shifting the median frequency of engagement from seven to eight or more logins may be considered a meaningful effect. As count data tend to be positively skewed, it was assumed that the primary outcome variable (‘frequency of engagement’) would follow a Poisson distribution. As the mean and median are almost identical for data that follow the Poisson distribution,^34^ power simulations (*N* = 1000) conducted in R indicated that 110 participants in the intervention arm and 440 participants in the control arm (reflecting the planned 1:4 randomisation ratio) would provide 90% power to detect a 14% increase (i.e. from seven to eight logins) in the mean frequency of engagement (incidence rate ratio (IRR) = 1.14). We judged this to be the minimum sample size required for the inferential analyses to proceed. However, our ‘stopping’ rule was pragmatic: as the number of users exceeded this threshold, we planned to include all users randomly allocated until randomisation stopped on 18 December 2018.

#### Descriptive statistics

Baseline characteristics of the two groups were compared using Chi-square tests or *t*-tests, as appropriate.

#### Inferential statistics

To address the first research question, data were first assessed for overdispersion (i.e. when the variance is greater than the mean). As data were overdispersed, a negative binomial (as opposed to a Poisson) distribution was specified. Group differences in the frequency of engagement were assessed using negative binomial regression analyses, with and without adjustment for time to first cigarette and CPD.

To address the second research question, group differences in quit success at one-month follow-up in the full sample (i.e. MES) were assessed using logistic regression analyses, with and without adjustment for time to first cigarette and CPD. We also conducted a sensitivity analysis, restricting the analyses to users who were successfully followed up (i.e. ‘follow-up only’ (FUO)).

#### Missing data

Participants with missing data on the primary outcome variable (‘frequency of engagement’) were excluded from all analyses. As per standards in tobacco monitoring surveys, such as the Smoking Toolkit Study,^35^ participants indicating that they smoked >100 CPD were treated as having missing entries for this variable. This had not been specified in the pre-registered analysis plan. Participants with missing data on time to first cigarette or CPD were excluded from all analyses including these variables.

#### Bayes Factors

Bayes Factors (BFs) and a Robustness Region (RR) for these BFs were calculated using an online calculator (http://www.lifesci.sussex.ac.uk/home/Zoltan_Dienes/inference/Bayes.htm) to examine whether the observed data provided evidence for the alternative (H1) or the null (H0) hypothesis. H1 was conservatively represented by a half-normal distribution, with the standard deviation of the distribution specified as the expected effect size described in the abovementioned power analysis (i.e. IRR = 1.14). The RR was notated as ‘RR (min, max)’, where min is the minimum effect size that leads to the same qualitative conclusion (i.e. good evidence for H1 over H0 if BF > 3; good evidence for H0 over H1 if BF < 1/3; and largely insensitive otherwise) and max is the maximum effect size that leads to the same conclusion.^36^

## Results

### Deviations from the pre-specified analysis plan

**Figure 2.**
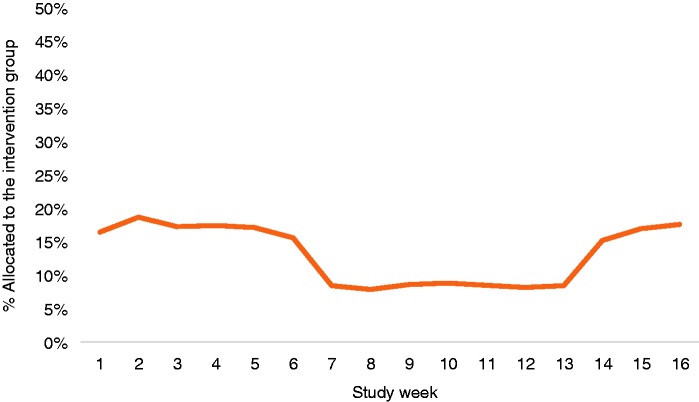
Plot of the proportion allocated to the intervention group by week at which users entered the study. The x-axis represents study week; the y-axis represents the proportion allocated to the intervention group.

Due to a coding error, the 1:4 randomisation ratio was not consistently applied throughout the study period. The observed randomisation ratio fluctuated between 8% and 19%, with clear breakpoints at weeks 7 and 14 (see Figure 2).

Due to another coding error, the pre-specified limit of counting a screen record as a new login only if at least 30 minutes of inactivity had lapsed was not imposed. Instead, a new login was automatically recorded each time a user accessed the app. As the ‘frequency of engagement’ variable did not have a temporal dimension embedded, it was not possible to derive the number of logins from the date of download until the one-month follow-up for each user. Instead, ‘frequency of engagement’ represents the total number of logins for each user tallied up until the date at which data were downloaded from the server (i.e. 29 March 2019). As users randomised earlier had a longer time period to accumulate logins, and the randomisation ratio fluctuated over the course of the study, we conducted an unplanned sensitivity analysis to examine whether the effect of group allocation on the frequency of engagement persisted across three cohorts, identified on the basis of the breakpoints in the plot in Figure 2 (i.e. weeks 1–6, weeks 7–13, weeks 14–16). In a second unplanned sensitivity analysis, we assessed whether the observed differences in the effect of group allocation by week in the study was driven by the fluctuating proportion of the number of highly engaged users (i.e. ‘power users’) across study arms. A ‘power user’ was defined as having engaged with the app ≥400 times, selected on the basis of a substantial drop in a histogram of the total frequency of engagement. In a third unplanned sensitivity analysis, we divided the number of logins for each user by the number of weeks in the study (i.e. average logins per week) and assessed whether the effect of group allocation persisted.

As a larger proportion of users from the intervention group were excluded following randomisation, we conducted a fourth sensitivity analysis repeating the primary analyses without the quit date eligibility criterion applied.

### Descriptive statistics

**Figure 3.**
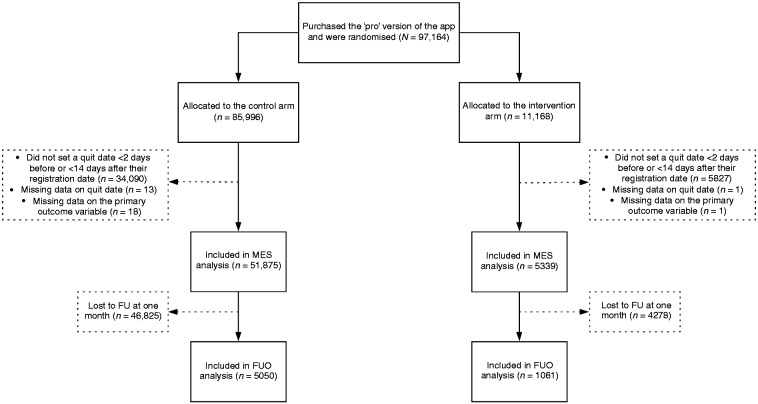
Participant flow chart. FU: follow-up; FUO: follow-up only; MES: missing equals smoking.

Figure 3 depicts the flow of participants. A total of 97,164 participants purchased the ‘pro’ version of the app and were randomised, with 88.5% allocated to the control group and 11.5% (11,168) allocated to the intervention group. Of these, 57,214 participants were eligible and were included in the analyses involving the full sample, with 90.7% (51,875) from the control group and 9.3% (5339) from the intervention group (see Table 1).

**Table 1.** Smoking characteristics in the full and follow-up only (FUO) samples.

	Full total(*N* = 57,214)	Full control(*n* = 51,875)	Full intervention(*n* = 5339)	*p* ^a^
Time to first cigarette, % (*n*)^b^				<.001
<5 min	23.9 (13,648)	19.3 (9999)	17.2 (917)	
5–30 min	37.7 (21,578)	19.0 (9880)	18.9 (1009)	
31–60 min	19.0 (10,889)	37.8 (19,605)	37.0 (1973)	
>60 min	19.1 (10,916)	23.6 (12,235)	26.5 (1413)	
Cigarettes per day, mean (SD)^c^	14.9 (9.1)	14.7 (8.9)	16.0 (11.4)	<.001
	FUO total(*N =* 6111)	FUO control(*n* = 5050)	FUO intervention(*n* = 1061)	*p* ^a^
Time to first cigarette, % (*n*)^d^				<.001
<5 min	20.4 (1245)	19.3 (973)	25.2 (267)	
5–30 min	38.5 (2351)	22.4 (1130)	36.9 (391)	
31–60 min	22.4 (1369)	38.8 (1960)	22.5 (239)	
>60 min	18.6 (1136)	19.3 (973)	15.4 (163)	
Cigarettes per day, mean (SD)^e^	15.8 (7.8)	15.6 (7.8)	16.5 (7.9)	<.001

^a^Differences between groups were compared using Chi-square tests, *t*-tests or Mood’s median test, as appropriate.

^b^Data on time to first cigarette were missing for 183 participants (intervention: 27, control: 156).

^c^Data on cigarettes per day were missing for 185 participants (intervention: 48, control: 137).

^d^Data on time to first cigarette were missing for 10 participants (intervention: 1, control: 9).

^e^Data on cigarettes per day were missing for 25 participants (intervention: 16, control: 9).

A total of 6,111 participants were included in the FUO analyses, with 9.7% (5050/51,875) from the control group and 19.9% (1061/5,339) from the intervention group (see Table 1). Compared with those who did not respond to the one-month follow-up survey, participants who did respond were less likely to smoke within <5 min of waking (χ^2^(3) = 77.4, *p* < .001) and smoked more CPD (*t*(8289.0) = –9.63, *p* < .001).

### Frequency of engagement

Results from the negative binomial regression analyses are displayed in Table 2. Being offered the addition of the supportive chatbot (median = 16, interquartile range (IQR) = 65.5), compared with the standard version of the Smoke Free app (median = 5, IQR = 22), was associated with a 107% increase in the frequency of engagement (*p* < .001). This association was not markedly attenuated when adjusting for time to first cigarette and CPD (*p* < .001).

**Table 2.** Effect of the chatbot on the frequency of engagement (*N* = 57,214).

	Frequency of engagementMedian (interquartile range)	IRR (95% CI)	*p*	IRR_adj_ (95% CI)^a^	*p*
Group					
Control	5 (22)	1.0		1.0	
Intervention	16 (65.5)	2.07 (1.97–2.17)	<.001	2.01 (1.92–2.11)	<.001
Time to first cigarette					
>60 min		–		1.0	
31–60 min		–		1.04 (0.99–1.09)	.08
5–30 min		–		0.89 (0.85–0.92)	<.001
<5 min		–		0.75 (0.71–0.78)	<.001
Cigarettes per day		–		1.03 (1.028–1.033)	<.001

^a^Participants with missing data on time to first cigarette and/or cigarettes per day (*n* = 267) were excluded from the adjusted analyses.

IRR: incidence rate ratio; CI: confidence interval

### Unplanned sensitivity analyses

**Figure 4.**
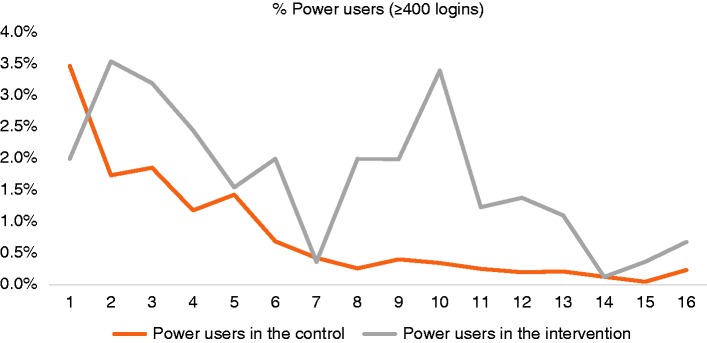
Plot of the proportion of ‘power users’ (i.e. participants who engaged with the app ≥400 times during the study period) by week of randomisation, split by study arm.

The effect of group allocation on the frequency of engagement in those randomised in weeks 1–6 (IRR_adj_ = 1.35, 95% confidence interval (CI) = 1.24–1.46, *p* < .001) and weeks 14–16 (IRR_adj_ = 1.29, 95% CI = 1.19–1.40, *p* < .001) was substantially attenuated. The effect in those randomised in weeks 7–13 (IRR_adj_ = 2.90, 95% CI = 2.70–3.11, *p* < .001) was substantially larger than that observed in the primary analysis in the full sample. This was partly driven by spikes in the proportion of ‘power users’ in the intervention group during weeks 7–13 (see Figure 4).

When dividing the number of logins by weeks in the study (i.e. average number of logins per week), the effect of group allocation on the frequency of engagement was similar to the primary analysis (IRR_adj_ = 2.02, 95% CI = 1.94–2.11, *p* < .001).

When repeating the primary analysis without the quit date criterion applied (*n* = 97,131), the effect of the chatbot on the frequency of engagement was slightly attenuated (IRR_adj_ = 1.90, 95% CI = 1.84–1.96, *p* < .001).

### Smoking cessation

Results from the logistic regression analyses are displayed in Table 3. In the MES analyses, participants offered the addition of the supportive chatbot (15.8%), compared with the standard version of the Smoke Free app (7.1%), had 2.44 times greater odds of being abstinent at the one-month follow-up survey (*p* < .001). This association was not substantially attenuated when adjusting for time to first cigarette and CPD (*p* < .001). In the FUO analyses, participants offered the addition of the supportive chatbot (79.5%), compared with the standard version of the Smoke Free app (73.3%), had 1.41 times greater odds of being abstinent at the one-month follow-up (*p* < .001). This association was not substantially attenuated when adjusting for time to first cigarette and CPD (*p* < .001).

**Table 3.** Effect of the chatbot on self-reported quitting success in the missing equals smoking and follow-up only analyses.

	MES (*N =* 57,214)					FUO (*N* = 6111)				
	Quitting success% (*N*)	OR (95% CI)	*p*	OR_adj_ (95% CI)^a^	*p*	Quitting success% (*N*)	OR (95% CI)	*p*	OR_adj_ (95% CI)^b^	*p*
Group										
Control	7.1 (3704)	1.0		1.0		73.3 (3704)	1.0		1.0	
Intervention	15.8 (844)	2.44 (2.25–2.64)	<.001	2.38 (2.19-2.58)	<.001	79.5 (844)	1.41 (1.20–1.66)	<.001	1.36 (1.16–1.61)	<.001
Time to first cigarette										
>60 min		–		1.0			–		1.0	
31–60 min		–		1.19 (1.08–1.31)	<.001		–		1.04 (0.87–1.24)	.68
5–30 min		–		1.00 (0.91–1.10)	.98		–		1.11 (0.94–1.32)	.22
<5 min		–		0.77 (0.70–0.86)	<.001		–		1.21 (0.98–1.49)	.08
Cigarettes per day		–		1.02 (1.01–1.02)	<.001		–		1.02 (1.02–1.03)	<.001

^a^Participants with missing data on time to first cigarette and/or cigarettes per day (*n* = 267) were excluded from the adjusted analyses.

^b^Participants with missing data on time to first cigarette and/or cigarettes per day (*n* = 15) were excluded from the adjusted analyses.

adj: adjusted; CI: confidence interval; FUO: follow-up only; MES: missing equals smoking; OR: odds ratio

When repeating the analyses without the quit date criterion applied, the effect of the chatbot on quit success was substantially attenuated in the MES analyses (adjusted odds ratio (OR_adj_) = 1.60, 95% CI = 1.51–1.69, *p* < .001) and no longer significant in the FUO analyses (OR_adj_ = 1.02, 95% CI = 0.92–1.13, *p* = .71).

### BFs

The calculation of BFs indicated that the data on the frequency of engagement provided substantial evidence for H1 (BF = >100). Setting the expected effect size to a value as low as 1.01 or as high as 1,000,000,000.00 did not enable us to draw a qualitatively different conclusion (all BFs >3). We also calculated BFs when representing H1 by a uniform distribution, iteratively changing the lower and upper bound of the expected effect size (i.e. 1–2, 2–3, etc.). This enabled us to draw a qualitatively different conclusion when the lower bound was set to 2 and the upper bound set to 3 (BF = 0.00).

## Discussion

### Principal findings

Our findings show that smokers allocated to receive the addition of the supportive chatbot engaged more frequently with the Smoke Free app than those allocated to receive the standard version of the app without the chatbot. The observed effect of the chatbot on engagement was large but fluctuated depending on the period of randomisation. A sensitivity analysis showed that this was partly driven by spikes in the proportion of self-selected ‘power users’ in the intervention group during particular periods of randomisation. In another sensitivity analysis regressing the average number of logins per week onto group allocation, the effect size was similar to the primary analysis in the full sample.

To account for biases due to loss to follow-up, we used both MES and FUO analyses. In the MES analyses, smokers who received the supportive chatbot had 2.38 times greater odds of quit success after adjusting for CPD and time to first cigarette. However, these odds were substantially attenuated in the FUO analyses (i.e. OR = 1.36). While the MES analyses may have biased effect sizes downwards if loss to follow-up occurred for reasons other than relapse to smoking, they may have biased effect sizes upwards if the intervention group were more likely to respond to the follow-up survey. Indeed, 19.9% of participants in the intervention group, versus 9.7% in the control group, responded to the follow-up survey. A true effect of the chatbot on quit success is expected to lie somewhere between the effects estimated in the MES and the FUO analyses. Moreover, in the sensitivity analysis repeating the analyses without applying the eligibility criterion of setting a quit date within the pre-specified time window, the effect of the chatbot on quit success was substantially attenuated in the MES analyses and no longer significant in the FUO analyses. This reduces confidence in the evidence and our findings should be interpreted with caution.

### Strengths and limitations

To our knowledge, this was the first study to quantify the added effect of an embedded conversational agent on engagement and effectiveness within a digital smoking cessation intervention. The popularity of the Smoke Free app (i.e. ∼3,000 new downloads per day) meant that this was a useful test bed for identifying features that promote engagement. Although the a priori power analysis indicated that at least 550 participants were required to detect a meaningful effect on engagement, >55,000 eligible participants were recruited into the study.

This study had important limitations. First, the calculation of BFs indicated that the observed data provided strong evidence for H1. However, the calculation of a Robustness Region for the BFs was not a useful exercise as we were unable to identify an inflection point at which our data no longer provided evidence for the alternative hypothesis. This was due to our decision to represent H1 by a half-normal, one-tailed distribution. When instead representing H1 by a uniform distribution, the BFs indicated that the observed data provided strong evidence for H1 up to an expected effect size of 2–3, at which our data provided evidence of H0.

Second, there were systematic baseline differences between groups in CPD and time to first cigarette. These differences can partly be explained by users being randomised prior to entering baseline characteristics, the fluctuating randomisation ratio across the study period, unequal exclusion of participants across study arms due to not setting a quit date within the pre-specified time window and unequal missingness in CPD across study arms. A greater number of users in the control group had missing data on CPD (intervention: 48; control: 137) and time to first cigarette (intervention: 27; control: 156). If more dependent users in the control group were less likely to complete the baseline assessment and less dependent users in the intervention arm set a quit date that did not fall within the pre-specified time window (and were excluded), this could have biased the control group estimates downwards. This limitation decreases the quality of the evidence. Future research should ensure that randomisation procedures are robust.

Third, as the number of logins for each user was tallied up without imposing a 30-minute time limit as cut-off, the absolute number of logins in the present study is likely to be inflated. We therefore caution against putting too much emphasis on the absolute frequency of engagement. Fourth, it is plausible that those allocated to the chatbot may have been more likely to go back and change their quit date after having interacted with the bot, which may have led to the exclusion of less engaged users from the primary analysis. This may serve as an explanation for the observed spikes in the proportion of ‘power users’ during weeks 7–13 of the study.

Fifth, this study was also limited by including only iPhone users, who on average tend to be more affluent than Android users.^37^ Due to funding restrictions, the chatbot was only available to iPhone users at the time of the study. Sixth, although power was not an issue given the large sample size, it should be noted that the a priori power analysis relied on a different model of smoking cessation, compared with that used in the present study, to determine what constitutes a meaningful increase in engagement with the Smoke Free app. Additional work is required to define what a meaningful increase in engagement may constitute across devices, subgroups of participants and models of smoking cessation. Seventh, the study sample was drawn from users who purchased the ‘pro’ version of the Smoke Free app, which may limit the generalisability of the findings to users who are willing to pay for a smoking cessation app. Eighth, there was substantial loss to follow-up, with a total of 10.7% of the overall sample responding to the one-month follow-up survey. Low follow-up rates are common in digital health research.^38^ If possible, researchers should hence consider contacting participants via multiple survey modalities (e.g. telephone, email, postcard) and incentivise survey completion as research shows that these strategies can greatly improve follow-up rates in online trials.^39–41^ Moreover, this study did not include an objective measure of quit success, which may have inflated cessation rates. However, risk of social desirability and false reporting is less of an issue for online studies with no face-to-face contact.^42^ Finally, data on age, sex and social grade were not captured as part of the app registration process and could hence not be included in the adjusted analyses.

### Implications and avenues for future research

For the purpose of the present study, it was assumed that the chatbot would have an additive effect on engagement. However, it is also plausible that the chatbot interacted synergistically (or antagonistically) with some or all of the other app components, meaning that their joint effect may have been greater (or smaller) than the sum of their separate effects. A factorial design is required to elucidate this (see Crane et al.^43^ for a recent example).

Based on the assumption that the chatbot has an additive effect on engagement, results from the present study can help to inform sample size calculations for future evaluation studies. Although the development and implementation of a chatbot within an existing app requires substantial expertise, time and financial resources, effects on engagement and effectiveness appear to be large. Future research should also endeavour to quantify the added effects of novel features that are relatively cheaper to implement (e.g. context-sensitive push notifications) on both engagement and effectiveness.

It should, however, be noted that the total frequency of engagement is not sufficient for successful behaviour change to occur; previous research has highlighted that engagement with particular app components (also referred to as the ‘depth of use’^18^), as opposed to ‘global’ engagement, is important for intervention effectiveness.^33^ Future research exploring the effect of embedded conversational agents on a broader range of indicators of behavioural (e.g. amount and depth of use) and experiential (e.g. attention, interest) engagement is hence warranted.

This study was unable to shed light on the potential working mechanisms of the chatbot. Qualitative methods, such as think aloud and semi-structured interview techniques, should be used to explore whether there is support for Mohr’s ‘Model of Supportive Accountability’,^23^ which posits that the addition of human (or by extension, human-like) support fosters a sense of supportive accountability to a trustworthy, benevolent and competent coach. Whether or not the chatbot increased engagement via other mechanisms of action (e.g. motivation to stay quit, perceived usefulness and personal relevance) should also be assessed.^18,20^

## Conclusion

The addition of a supportive chatbot powered by AI to a popular smoking cessation app more than doubled engagement with the app. In view of very low follow-up rates, there is low quality evidence that the addition also increased self-reported smoking cessation at a one-month follow-up.

## Supplemental Material

DHJ880676 Supplemental Material - Supplemental material for Does the addition of a supportive chatbot promote user engagement with a smoking cessation app? An experimental study Click here for additional data file. Supplemental material, DHJ880676 Supplemental Material for Does the addition of a supportive chatbot promote user engagement with a smoking cessation app? An experimental study by Olga Perski, David Crane, Emma Beard and Jamie Brown in Digital Health

## Data Availability

The dataset and R code used in the current study are available from the corresponding author on reasonable request.
