# Corrigendum to Does the Addition of a Supportive Chatbot Promote User Engagement with a Smoking Cessation App

**DOI:** 10.1177/2055207620930958

**Published:** 2020-05-22

Perski O, David C, Emma B, and Jamie B. “Does the Addition of a Supportive Chatbot Promote User Engagement with a Smoking Cessation App? An Experimental Study.” *DIGITAL HEALTH*. DOI: 10.1177/2055207619880676

In this article, the values in Table 1 were incorrect. The correct table is mentioned as below:

**Table 1.** Smoking characteristics in the full and follow-up only (FUO) samples.

	Full Total (*N* = 57,214)	Full Control (*n* = 51,875)	Full Intervention (*n* = 5,339)	p*
Time to first cigarette, % (N)^a^				<.001
>60 minutes	19.1% (10,916)	19.3% (9,999)	17.2% (917)	
31-60 minutes	19.0% (10,889)	19.0% (9,880)	18.9% (1,009)	
5-30 minutes	37.7% (21,578)	37.8% (19,605)	37.0% (1,973)	
<5 minutes	23.9% (13,648)	23.6% (12,235)	26.5% (1,413)	
Cigarettes per day, mean (*SD*)^b^	14.9 (*9.1*)	14.7 (*8.9*)	16.0 (*11.4*)	<.001

	FUO Total (*N =* 6,111)	FUO Control (*n* = 5,050)	FUO Intervention (*n* = 1,061)	p*
Time to first cigarette, % (N)^c^				<.001
>60 minutes	18.6% (1,136)	19.3% (973)	15.4% (163)	
31-60 minutes	22.4% (1,369)	22.4% (1,130)	22.5% (239)	
5-30 minutes	38.5% (2,351)	38.8% (1,960)	36.9% (391)	
<5 minutes	20.4% (1,245)	19.4% (978)	25.2% (267)	
Cigarettes per day, mean (*SD*)^d^	15.8 (*7.8*)	15.6 (*7.8*)	16.5 (*7.9*)	<.001

*Note.* ^a^Data on time to first cigarette were missing for 183 participants (intervention: 27, control: 156). ^b^Data on CPD were missing for 185 participants (intervention: 48, control: 137). ^c^Data on time to first cigarette were missing for 10 participants (intervention: 1, control: 9). ^d^Data on CPD were missing for 25 participants (intervention: 16, control: 9). *Differences between groups were compared using Chi-square tests or *t*-tests, as appropriate.

