# Supplementary material for: Does the addition of a supportive chatbot promote user engagement with a smoking cessation app? An experimental study
Source: Digit Health. 2019 Sep 30;5:2055207619880676. doi: 10.1177/2055207619880676 (PMC6775545; doi:10.1177/2055207619880676)
Supplement: DHJ880676 Supplemental Material - Supplemental material for Does the addition of a supportive chatbot promote user engagement with a smoking cessation app? An experimental study [file DHJ880676_Supplemental_Material.pdf]

Table 2. Behaviour change techniques (BCTs) included in the Smoke Free app

BCT Code and Label	BCT Present?*	
	Control	Intervention
Specific focus on behaviour (B) and addressing motivation (M)		
BM1. Provide information on consequences of smoking and smoking cessation		✓
BM2. Boost motivation and self-efficacy	✓	✓
BM3. Provide feedback on current behaviour	✓	✓
BM4. Provide rewards contingent on successfully stopping smoking	✓	✓
BM5. Provide normative information about others' behaviour and experiences		
BM6. Prompt commitment from the client here and then	✓	✓
BM7. Provide rewards contingent on effort or progress	✓	✓
BM8. Strengthen ex-smoker identity		✓
BM9. Identify reasons for wanting and not wanting to stop smoking		✓
BM10. Explain the importance of abrupt cessation		✓
BM11. Measure expired-air carbon monoxide (CO) concentration		
Specific focus on behaviour (B) and maximising self-regulatory capacity/skills (S)		
BS1. Facilitate barrier identification and problem solving		✓
BS2. Facilitate relapse prevention and coping		✓
BS3. Facilitate action planning/develop treatment plan	✓	✓
BS4. Facilitate goal setting	✓	✓
BS5. Prompt review of goals	✓	✓
BS6. Prompt self-recording	✓	✓
BS7. Advise on changing routine		✓
BS8. Advise on environmental restructuring		✓
BS9. Set graded tasks		
BS10. Advise on conserving mental resources		✓
BS11. Advise on avoiding social cues for smoking		✓
Promote adjuvant activities (A)		
A1. Advise on stop-smoking medication		✓
A2. Advise on/facilitate use of social support		✓
A3. Adopt appropriate local procedures to enable clients to obtain free medication		
A4. Ask about experiences of stop smoking medication that the smoker is using		
A5. Give options for additional and later support		
General aspects of the interaction (R) focusing on the delivery of the intervention (D)		
RD1. Tailor interactions appropriately		✓
RD2. Emphasise choice		✓
General aspects of the interaction (R) focusing on information gathering (I)		
RI1. Assess current and past smoking behaviour	✓	✓
RI2. Assess current readiness and ability to quit		

RI3. Assess past history of quit attempts		
RI4. Assess withdrawal symptoms		
General aspects of the interaction (R) focusing on general communication (C)		
RC1. Build general rapport	✓	✓
RC2. Elicit and answer questions		
RC3. Explain the purpose of CO monitoring		
RC4. Explain expectations regarding treatment programme		✓
RC5. Offer/direct towards appropriate written materials		✓
RC6. Provide information on withdrawal symptoms		✓
RC7. Use reflective listening		
RC8. Elicit client views		
RC9. Summarise information/confirm client decisions		
RC10. Provide reassurance		✓

Note. * BCT presence is indicated by a tick.
